# Synergistic Effects of Human Milk Nutrients in the Support of Infant Recognition Memory: An Observational Study

**DOI:** 10.3390/nu7115452

**Published:** 2015-11-03

**Authors:** Carol L. Cheatham, Kelly Will Sheppard

**Affiliations:** 1 Nutrition Research Institute, University of North Carolina at Chapel Hill, Kannapolis, NC 28081, USA; 2 Department of Psychology & Neuroscience, University of North Carolina at Chapel Hill, Chapel Hill, NC 27599, USA; kelly_sheppard@unc.edu

**Keywords:** breastmilk, DHA, choline, lutein, recognition memory, infant cognition, synergy, electrophysiology, nutrition

## Abstract

The aim was to explore the relation of human milk lutein; choline; and docosahexaenoic acid (DHA) with recognition memory abilities of six-month-olds. Milk samples obtained three to four months postpartum were analyzed for fatty acids, lutein, and choline. At six months, participants were invited to an electrophysiology session. Recognition memory was tested with a 70–30 oddball paradigm in a high-density 128-lead event-related potential (ERP) paradigm. Complete data were available for 55 participants. Data were averaged at six groupings (Frontal Right; Frontal Central; Frontal Left; Central; Midline; and Parietal) for latency to peak, peak amplitude, and mean amplitude. Difference scores were calculated as familiar minus novel. Final regression models revealed the lutein X free choline interaction was significant for the difference in latency scores at frontal and central areas (*p* < 0.05 and *p* < 0.001; respectively). Higher choline levels with higher lutein levels were related to better recognition memory. The DHA X free choline interaction was also significant for the difference in latency scores at frontal, central, and midline areas (*p* < 0.01; *p* < 0.001; *p* < 0.05 respectively). Higher choline with higher DHA was related to better recognition memory. Interactions between human milk nutrients appear important in predicting infant cognition, and there may be a benefit to specific nutrient combinations.

## 1. Introduction

On a molecular level, nutrients appear to be integral to brain development and subsequent cognition. Nonetheless, empirical evidence of a relation between nutrient intake and brain function has been elusive. The difficulty in documenting nutritional effects on brain could be because most research is focused on a single nutrient. Nutrients do not exist in isolation nor is consumption limited to one type of food. Thus, nutrients may work synergistically in the brain. We tested the effects of choline, docosahexaenoic acid (DHA), and lutein on recognition memory in six-month-old infants using an electrophysiology paradigm known as event-related potentials (ERP).

*Early* choline status has been shown to be important for *later* adult memory in animal models [1]. Given the distal nature of the effects, it is not surprising that when *early* choline was tested in relation to *early* cognition, the results were mixed with some researchers not finding definitive effects [2,3] and others reporting positive effects [4]. Indeed, in data from Project Viva, no relation was found between maternal intake of choline and cognition at three years [5], but associations emerged at seven years [6]. Others have found effects of concurrent choline on cognition in adults [7]. Evidence from animal models indicates that the integrity of the hippocampus and by extension, visuospatial memory, is related to choline intake. Importantly, choline is co-localized with DHA in the brain and liver.

Fatty acid researchers also have found mixed results when supplementing infant diets with DHA; fewer than 40% of trials evidence an effect on cognition when infants are born full-term [8], although researchers working with infants born pre-term have found more consistent results [9,10] albeit short-term [11]. Nonetheless, DHA is prevalent in the structures that underlie cognitive abilities, specifically, the eye, the hippocampus, and the frontal brain areas. There is evidence to suggest that DHA supplementation in infancy improves problem-solving [12] and visual acuity [13] in infancy and hippocampal-based cognitive outcomes in toddlerhood [14]. Suggestively, researchers have shown the importance of controlling for DHA when studying environmental contaminants, as DHA is protective against the negative effects of toxins, such as mercury, on the brain and subsequent cognition [15,16].

In the aging primate eye, lutein has been associated with reduced damage to the macula from blue light [17], and in adults there is an association between lutein and cognition [18,19]. In the developing eye, macular pigment optical density increases as serum lutein increases [20]. Lutein is deposited into the macula (which is the most immature structure of the eye at birth) concurrent with the development of visual acuity, which occurs across the first year of life. However, it is not known if the two are related: very little work has been done on the effects of lutein early on. Animal models suggest that lutein is important to the development of the eye [21] as is DHA. In one study of the association between plasma lutein and cognition in young children (five to six years old), no relation was found [22]. However, the design of the study included lutein as a single nutrient, as is common practice.

As noted, nutrients most likely work together to exact effects, and small hints at synergistic effects have emerged. In tests of delayed recall, Johnson [19] showed that delayed recall was better in elderly participants who were supplemented with DHA and lutein, relative to those who were supplemented with DHA or lutein singularly. In the liver, choline is necessary for the release of DHA stores into the bloodstream. Moreover, DHA and lutein are transported to the brain by phospholipid-rich lipoproteins [23]. For these reasons, we hypothesize that the effects of nutrition on recognition memory will be greater based on two nutrients, as opposed to a single nutrient.

## 2. Experimental Section

### 2.1. Participants

One hundred five exclusively breastfed 6-month-old infants participated in the context of a larger study conducted in the southeastern United States between 2010 and 2012. All but two of the 105 infants (98.1%) were 100% breastfed; the two others were supplemented with no more than 10% formula. The mothers were under 45 years of age, had unremarkable pregnancies, and gave birth to healthy, single infants at term. The sample was 82% Caucasian and 49% male. The data presented here are a secondary analysis.

Of those who came in for the ERP session, 67 participants (mean age 6.1 months (mo), sd = 0.9) provided good data; 2 participants did not tolerate the net; 3 sessions had no data due to technical difficulties; 6 sessions were stopped due to fussing and crying; and 24 participants did not provide sufficient clean data (defined as a minimum of 10 artifact free segments for each condition). This dropout rate is within that typically seen in infant ERP studies [24,25]. The analyses include those from the larger study who had clean ERP data as well as milk choline, lutein, and DHA data (*n* = 55).

### 2.2. Study Design

The participants were invited to the laboratory where the study was explained to them in detail. All gave written informed consent for themselves and their infants in accordance with the Institutional Review Board (09-1869; current approval date 31 August 2015). This research study is registered at ClinicalTrials.gov (NCT01942434).

#### 2.2.1. Milk Collection

In the context of the larger study, when their infants were 3–5 months of age, mothers collected a sample of their first morning milk by emptying one entire breast. The milk was then agitated, aliquoted, and transported to the lab on ice. Samples were immediately frozen at −20 °C. Frozen milk samples were transferred to the Nutrition Obesity Research Center (NORC) on dry ice where they were then stored at −80 °C until lipids were extracted and saponified. For these analyses, the remaining milk was shipped on dry ice to an independent laboratory where lutein, free choline, and betaine were quantified.

#### 2.2.2. Fatty Acid Quantification

At the UNC at Chapel Hill NORC, milk samples were analyzed for fatty acid content. Lipids were extracted from the breast milk samples using the method of Bligh and Dyer [26]. The lower (chloroform) phase was transferred to a clean tube and evaporated to dryness under nitrogen. The residual lipids were saponified and the fatty acids trans-methylated by sequential 1 mL addition of 4.25 % NaOH in CHCl3:MeOH (2:1, *v*/*v*) and 1N HCl in saline [27]. The samples were mixed vigorously then centrifuged at 1500 *g* for 5 min. The lower phase containing the fatty acid methyl esters was carefully transferred to a clean, dry tube and evaporated to dryness under nitrogen. Fatty acid methyl esters were then re-suspended in 50 L undecane, and analyzed using capillary gas chromatography (GC). Fatty acid methyl esters were analyzed by Fast GC on a Perkin Elmer AutoSystem XL Gas Chromatograph (Shelton, CT), split injection, with helium as the carrier gas. The methyl esters were separated on a capillary column coated with 70% cyanopropyl polysilphenylene—siloxane (10 m × 0.1 mm ID-BPX70 0.2 m; SGE, Austin, TX, USA); injector 240 °C and detector 280 °C. Data were analyzed with the Perkin Elmer Totalchrom Chromatography Software, version 6.2. Heptadecanoic acid (17:0) was added to the samples as an internal standard to correct for recovery and quantitation. Individual fatty acids were identified by comparing their retention with authentic standards (Nu Chek Prep, Elysian, MN, USA).

#### 2.2.3. Choline and Betaine Quantification

Free choline and betaine were assayed in the Innis lab as detailed in Innis and Hassman [28]. Twenty μL of milk was transferred to a 1.5 mL Eppendorf tube containing 10 μL of internal standards, deuterium labeled free choline, and vortexed. 60 μL of methanol was added and the samples were centrifuged (14000× *g* for 10 min). The supernatant was transferred and diluted 1/5 in an autosampler vial containing mobile phase. Chromatographic separation was performed isocratically with 4 μL injection of sample using an Agilent Zorbax silica column (2.1 × 150 mm with 5 μm pore size) and a mobile phase consisting of 20% 15 mmol/L ammonium formate, 0.1% formic acid in H20, and 80% acetonitrile.

#### 2.2.4. Lutein Quantification

Lutein content of the milk was determined using a liquid:liquid extraction method as described by Yuhas and colleagues [29]. One mL of ethanol was added to 1 mL of a sample of human milk and vortexed for 10 seconds. Two mL of Hexane:THF (80:20) were added to the samples and the tubes were shaken for 15 min. The tubes were centrifuged for 15 min at 3500 rpm and the organic layer was transferred to a new test tube. This extraction was repeated, and the organic phases were combined. These combined organic phases were then extracted with 2 mL of ethanol: H_2_O (90:10) and briefly vortexed, shaken for 10 min, and centrifuged for 5 min at 3500 rpm. The aqueous layer was transferred to a new test tube, and the extraction was repeated with the aqueous phases combined. The combined aqueous phases were dried under nitrogen and heat (30 °C). Standard solutions of lutein in ethanol were simultaneously carried through the extraction protocol beginning with the addition of 1 mL water and the hexane: THF step. Then, the sample and standard extracts were dissolved in 1 mL mobile phase (MeOH: MTBE, 85:15, *v*:*v*). Samples were vortexed and allowed to stand until clear. Samples were transferred to amber vials for HPLC analyses, leaving behind any insoluble material. The samples were run on an Agilent 1100 Series coupled with a Variable Wavelength Detector. Chromatographic separation was performed using a YMC C30 carotenoid column (5 μm, 4.6 × 250 mm) and a mobile phase gradient of Methanol: MTBE (85:15) to (70:30). Analysis was conducted with a 100 µL injection, a flow rate of 1 mL/min and detector wavelength of 445 nm. Total run time was 30 min. An external standard curve was prepared using chromatograms of extracted standard solutions.

#### 2.2.5. Electrophysiology Methods

In a protocol designed to test recognition memory, we recorded event-related potentials (ERP; electroencephalogram aka EEG time-locked to stimuli presentation) and eye movements. To enable recording, the participant was fitted with a 128-sensor Geodesic Sensor Net (GSN; Electrical Geodesic, Inc. (EGI), Eugene, OR, USA); data were recorded from 124 sensors. Measurements were obtained of the infant’s head to insure the proper size net was used, but also to insure proper placement of the vertex, mastoid, and other landmark sensors. Application of the net required approximately 15 min and was well tolerated by the infants (only 2 refused to wear the net). Impedances were checked and corrected if necessary to below 50 kΩ, and the net was connected to a NetAmps 300 (Electrical Geodesic, Inc., Eugene, OR, USA). The participant was seated on the caregiver’s lap 45 cm from a 19” Dell monitor in the participant room (separate from the control room). A Sony DCR-HC52 camera, positioned under the monitor and time-locked to the data being acquired, recorded the participant’s face during presentation of the pictures. Stimuli were presented by EPrime 2.0 software (Psychology Software Tools, Inc., Sharpsburg, PA, USA). Data were digitized at 250 Hz and stored by NetStation 4.4.2 (Electrical Geodesic, Inc., Eugene, OR, USA) on a Mac. During recording the data were referenced to a single point at the vertex. The EGI system used a single-clock to time-lock the presentation of the stimuli with the EEG continuous recording and the video capture.

#### 2.2.6. Stimuli

The stimuli computer displayed pictures of wooden toys gleaned from the internet. All pictures occupied the middle 50% of an 18 cm square on a white background with a 10 cm black border and presented at a viewing angle of 68 degrees. All participants were shown the same picture 12 times for 1500 ms each with an inter-trial interval (ITI) of 500 ms to familiarize them with the standard picture. The familiarization period was followed by the presentation of 100 pictures randomly ordered by EPrime and including the standard, familiar image (70%) and 30 trial-unique novel images (30%) [30] for 1500 ms separated by inter-stimuli intervals (500 ms) of randomly varied length (±0 to 200 ms). The novel images elicit reliable negative deflections (Nc) that peak approximately 600 ms after the stimulus presentation in 6-month-olds [31]. Caregivers were instructed in techniques to redirect the infant’s attention to the screen, if needed, to ensure the infant viewed as many pictures as possible. Lights were off during the procedure.

### 2.3. Data Reduction and Analyses

#### 2.3.1. Nutrients

Nutrient quantification data were visually examined for anomalies. To ensure that the results were not unduly influenced by a single data point and as is the convention [32], outliers (datapoint >3 SD above or below the mean) were removed from the dataset: one betaine (7.5 SD above the mean), two choline (3.2 and 5.2 SD above the mean), and one DHA (5.2 SD above the mean) datapoints were excluded.

#### 2.3.2. Electrophysiological Data

Recorded files were individually assessed for gaze direction based on pupillary reflection. Videos were segmented by trial, and segments were collected into three video streams based on condition (habituation, novel unique, familiar). The videos were opened, in turn, in iMovie ‘09 (Apple, Inc. Dolby Laboratories, San Francisco, CA, USA) and partitioned into segments each equal to the presentation of one trial. Trial segments were individually assessed for status of the participant’s gaze, and the status was noted on the event list. Trials in which the participant was not attending to the stimulus were subsequently marked as “bad” in the NetStation file. This process was repeated for each participant.

EEG waveforms were visually assessed for anomalies. Data were then subjected to a 30 Hz lowpass filter and segmented into 1700 ms segments (200 ms before and 1500 ms after stimulus onset). Bad channels were detected by the software using a moving average of 150 ms and a threshold of 250 μV. Segments that included more than 13 (>10%) bad channels were rejected. Data were baseline corrected using the mean voltage during the 200 ms preceding stimulus onset. The remaining segments were assessed for artifacts, and when necessary, individual channels were replaced using spherical spline interpolation. Trials were averaged within condition (novel, familiar). Across participants, the average number of trials viewed and producing clean data were 13 for novel unique (43%; range 10–30) and 27 for familiar (39%; range 10–66). Participants’ data were averaged together by condition for a grand mean.

**Figure 1:**
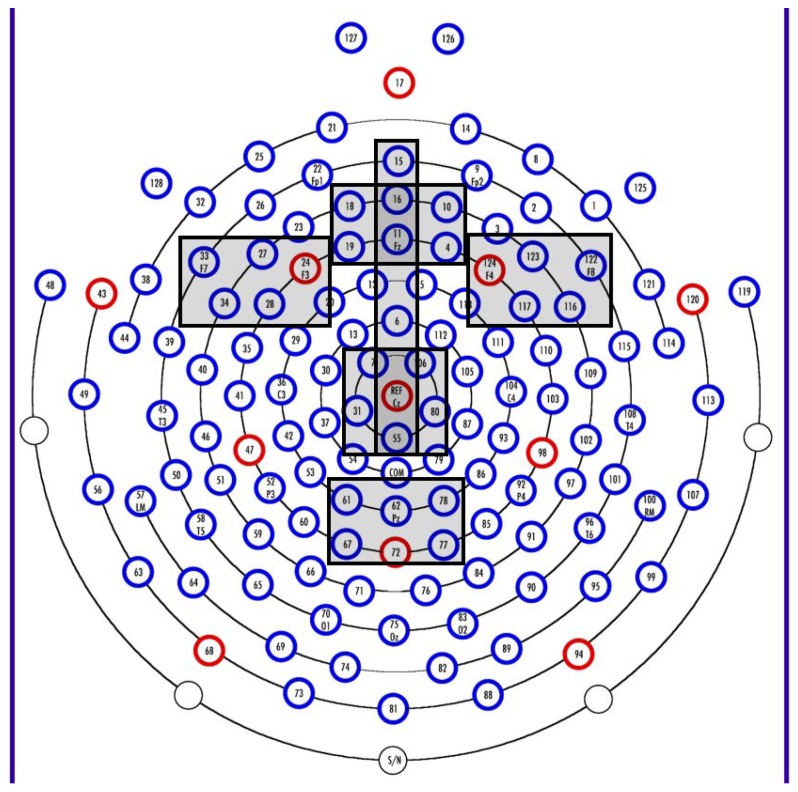
Sensor map showing the clusters used in analyses: FrontalZ (4, 10, 11, 16, 18, 19), FrontalL (24, 27, 28, 34, 33), FrontalR (116, 117, 122, 123, 124), CentralZ (7, 31, 55, 80, 106, REF), ParietalZ (61, 62, 67, 72, 77, 78), and Midline (6, 11, 15, 16, 55, REF).

The grand mean file was visually examined, and a negative deflection window of interest was chosen at 200 to 900 ms from stimulus onset. Visual inspection of the continuous data and review of the literature e.g., [33] provided support for sensor clusters of interest. Data were averaged across sensor groups as follows (see Figure 1): FrontalZ (4, 10, 11, 16, 18, 19), FrontalL (24, 27, 28, 34, 33), FrontalR (116, 117, 122, 123, 124), CentralZ (7, 31, 55, 80, 106, REF), ParietalZ (61, 62, 67, 72, 77, 78), and Midline (6, 11, 15, 16, 55, REF). Clusters were individually assessed for mean amplitude, peak amplitude, and latency to peak amplitude within the 200 to 900 ms window for each condition using NetStation 4.5.4 (Electrical Geodesic, Inc., Eugene, OR, USA). In SAS 9.2 (SAS Institute, Inc., Cary, NC, USA), difference variables were computed for each variable from each cluster by subtracting data for novel unique from data for familiar data.

#### 2.3.3. Data Analyses

Data met assumptions of normality and were analyzed in full model multivariate regressions using the three nutrients (DHA, choline, lutein) and the interactions to predict mean amplitude, peak amplitude, and latency to peak amplitude for each cluster using SAS 9.2. Data were then subjected to multivariate stepwise regressions to explore potential models of import. Significant results were followed up in reduced models. In addition, significant interactions were modeled using simple slopes analysis, which takes a low value of a variable (one standard deviation below the mean), the mean of a variable, and a high value of a variable (one standard deviation above the mean), and determines whether or not the slope changes significantly with respect to the second variable. The simple slopes procedure provides an analysis of how the variables interact by plotting the direction and rate of change at each level of the first variable [34].

## 3. Results

Data were examined to ensure that assumptions were met for regression analyses. Maternal education (as a proxy for socioeconomic status), head circumference, and infant age were tested as potential covariates by regressing the outcome variables on to each one in turn. It was determined that no covariates were necessary (all *p* > 0.05). Demographics as well as means and ranges of the nutrient levels are in Table 1. The data were then subjected to full model multivariate regressions that account for multiple comparisons within the analyses (Table 2). No predictors (choline, lutein, DHA, interactions) were significant at the *p* < 0.05 level for mean amplitude or peak amplitude. Latency at the central leads was predicted by choline (*p* < 0.01) and the lutein X choline interaction (*p* < 0.05). No other instances of significance were found.

**Table 1:** Human milk nutrient composition determined from an aliquot of milk expressed from one whole breast (*i.e.*, containing fore- and hind-milk) at 3–5 mo after parturition. Infant age at sample was computed using the n with complete nutrient data.

	N	Minimum	Maximum	Mean	SD
Infant age at sample (mo)	67	3.0	5.8	3.7	0.63
Infant age at test (mo)	67	5.9	6.4	6.1	0.09
Maternal education (yrs)	67	12	20	16.4	1.63
Head circumference (6 mo; cm)	67	41.0	46.5	43.4	1.18
DHA (g/100g)	61	0.08	1.07	0.26	0.18
Free choline (umol/L)	60	23.0	326.9	158.4	68.85
Lutein (mcg/L)	62	0.0	52.6	18.4	14.85

DHA: docosahexaenoic acid; SD: standard deviation; yrs: years.

**Table 2:** Results of the full model multivariate regression predicting the difference in latency to peak amplitude of the negative deflection seen between 200 and 900 ms when viewing novel *vs.* familiar stimuli.

Lead Grouping	Variable	β	SE	*t*-Value	*p*-Value	Model R-Square
Frontal Z	Intercept	−23.11	144.14	−0.16	0.87	0.04
	DHA	2.19	4.51	0.49	0.63	
	Choline	0.58	0.74	0.78	0.44	
	Lutein	−1.16	6.23	−0.18	0.86	
	DHA X Choline	−0.02	0.03	−0.82	0.41	
	DHA X Lutein	0.02	0.07	0.24	0.81	
	Lutein X Choline	−0.001	0.03	−0.02	0.98	
Frontal Right	Intercept	−144.48	152.55	−0.95	0.34	0.14
	DHA	5.55	4.77	1.16	0.25	
	Choline	1.57	0.79	2.00	0.05 *	
	Lutein	−6.77	6.68	−1.01	0.32	
	DHA X Choline	−0.04	0.03	−1.69	0.10	
	DHA X Lutein	0.04	0.07	0.55	0.59	
	Lutein X Choline	0.02	0.03	0.52	0.60	
Frontal Left	Intercept	−171.34	127.68	−1.34	0.19	0.21
	DHA	5.00	3.99	1.25	0.22	
	Choline	1.12	0.66	1.84	0.07 ^	
	Lutein	6.86	5.59	1.23	0.23	
	DHA X Choline	−0.04	0.02	−1.79	0.08 ^	
	DHA X Lutein	−0.01	0.06	−0.17	0.87	
	Lutein X Choline	−0.03	0.03	−0.98	0.33	
Central Z	Intercept	−307.51	135.06	−2.28	0.03 *	0.30
	DHA	3.27	4.22	0.77	0.44	
	Choline	1.90	0.70	2.73	0.009 **	
	Lutein	9.77	5.92	1.65	0.11	
	DHA X Choline	−0.04	0.02	−1.72	0.09 ^	
	DHA X Lutein	0.06	0.06	0.93	0.36	
	Lutein X Choline	−0.06	0.03	−2.12	0.04 *	
Midline	Intercept	−125.34	133.78	−0.94	0.35	0.13
	DHA	3.02	4.18	0.72	0.47	
	Choline	1.01	0.69	1.46	0.15	
	Lutein	4.28	5.86	0.73	0.47	
	DHA X Choline	−0.03	0.02	−1.25	0.22	
	DHA X Lutein	0.01	0.06	0.22	0.82	
	Lutein X Choline	−0.03	0.03	−0.88	0.38	
Parietal Z	Intercept	−46.23	188.45	−0.25	0.81	0.08
	DHA	−5.77	5.89	−0.98	0.33	
	Choline	−0.01	0.97	−0.01	0.99	
	Lutein	10.98	8.26	1.33	0.19	
	DHA X Choline	0.23	0.03	0.69	0.49	
	DHA X Lutein	0.26	0.09	0.29	0.77	
	Lutein X Choline	−0.07	0.04	−1.59	0.12	

* Significant at *p* < 0.05; ^ Trending to significance at *p* < 0.10. DHA: docosahexaenoic acid; R-squared: coefficient of determination; SE: standard error; ** Significant at *p* < 0.01.

Because the sample size was small, modeling was undertaken to insure that reduced models of significance were not overlooked. Data were entered into multivariate stepwise regressions to account for multiple comparisons using all the predictors (choline, lutein, DHA, and interactions; Table 3). No variables predicted mean amplitude or peak amplitude at any location cluster. Latency at left frontal was predicted by DHA X choline interaction (*p* < 0.01). DHA X lutein also entered the model at *p* < 0.10. The model accounted for 13.5% of the variance. Latency was also predicted at the central leads by choline (*p* < 0.05), lutein (*p* = 0.001), and lutein X choline (*p* < 0.001). This model accounted for 24% of the variance.

Significance in the model-building exercise was followed up with analyses of the reduced models. DHA, choline, and the interaction were used to predict latency to peak amplitude (Table 4). Latency at right frontal leads was predicted by choline (*p* < 0.05) and the DHA X choline interaction showed a trend (*p* < 0.10). The model accounted for 11% of the variance. Left frontal latency was also predicted by DHA (*p* < 0.05) and the DHA X choline interaction (*p* < 0.01) with 18% of the variance explained. For latency at the central leads, both nutrients predicted (DHA, *p* < 0.01; choline, *p* < 0.05) as well as the interaction (DHA X choline, *p* < 0.001) with 21% of the variance explained. Finally, latency along the midline was predicted by DHA X choline (*p* < 0.05) with a DHA trending toward prediction (*p* < 0.10). This final model predicted 12% of the variance.

**Table 3:** Model-building results from multivariate stepwise regressions predicting the difference in latency to peak amplitude of the negative deflection seen between 200 and 900 ms when viewing novel *vs.* familiar stimuli. Variables were set to enter the equation if *p* < 0.15.

Lead Grouping	Variable	β	SE	F-Value	*p*-Value	Model F	Model p	Model R-sq
Frontal Left	Intercept	85.43	35.7	5.73	0.02 *	4.04	0.02 *	0.13
	DHA X Choline	−0.02	0.007	7.88	0.007 **			
	DHA X Lutein	0.05	0.03	3.13	0.08 ^			
Central Z	Intercept	−266.55	96.24	7.67	0.008 **	5.26	0.003 **	0.24
	Choline	1.13	0.53	4.61	0.04 *			
	Lutein	15.66	4.46	12.35	0.0009 ***			
	Lutein X Choline	−0.09	0.02	14.30	0.0004 ***			

*** Significant at *p* < 0.001; ** Significant at *p* < 0.01; * Significant at *p* < 0.05; ^ Trending toward significance at *p* < 0.1; DHA: docosahexaenoic acid; SE: standard error.

**Table 4:** Reduced model exploration of the significant interactions from the model-building analyses. DHA, choline, and DHA X choline were used to predict the difference in latency to peak amplitude of the negative deflection seen between 200 and 900 ms when viewing novel *vs.* familiar stimuli. Only lead groupings showing a predictor at the *p* < 0.15 level in previous analyses were analyzed.

Lead Grouping	Variable	β	SE	F-Value	*p*-Value	Model F	Model p	Model R-sq
Frontal Right	Intercept	−229.54	127.45	−1.80	0.08 ^	2.02	0.12	0.11
	DHA	5.38	3.46	1.55	0.12			
	Choline	1.75	0.77	2.28	0.03 *			
	DHA X Choline	−0.04	0.02	−1.92	0.06 ^			
Frontal Left	Intercept	−117.69	106.95	−1.10	0.27	3.66	0.02 *	0.18
	DHA	6.81	2.90	2.35	0.02 *			
	Choline	1.05	0.64	1.63	0.11			
	DHA X Choline	−0.05	0.02	−2.75	0.008 **			
Central Z	Intercept	−312.00	117.62	−2.65	0.01 *	4.54	0.007 **	0.21
	DHA	9.89	3.19	3.10	0.03 *			
	Choline	1.73	0.71	2.44	0.02 *			
	DHA X Choline	−0.07	0.02	−3.44	0.001 **			
Midline	Intercept	−118.24	110.56	−1.07	0.29	2.27	0.09 ^	0.12
	DHA	5.38	3.00	1.79	0.08 ^			
	Choline	0.94	0.67	1.40	0.17			
	DHA X Choline	−0.04	0.02	−2.18	0.03 *			

*** Significant at *p* < 0.001; ** Significant at *p* < 0.01; * Significant at *p* < 0.05; ^ Trending toward significance at *p* < 0.10; DHA: docosahexaenoic acid.

**Figure 2:**
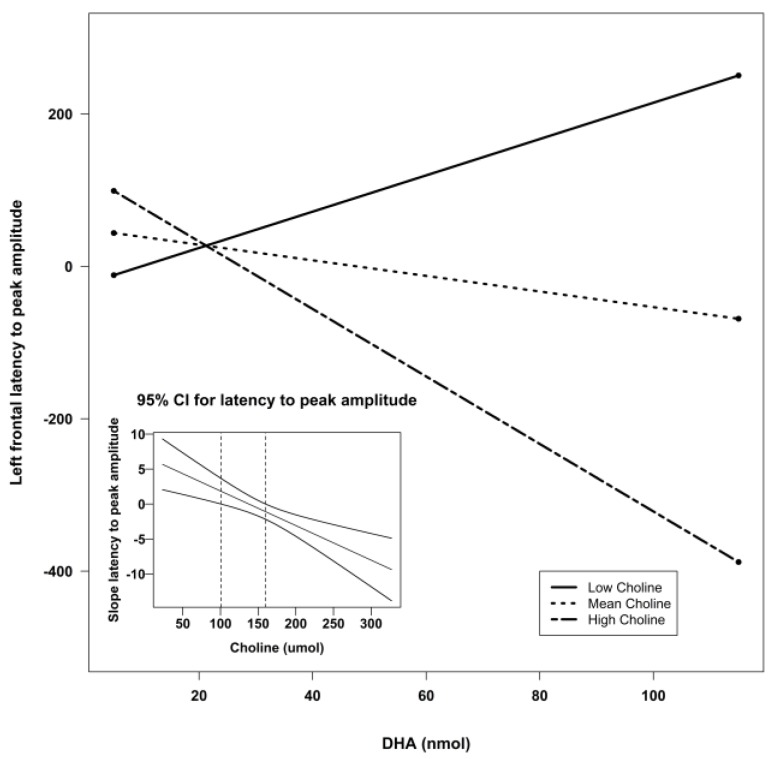
Simple slopes model of DHA by choline interaction for latency to peak amplitude at the left frontal sensors. Negative numbers are indicative of better recognition. The figure depicts the effect of DHA at high, mean, and low levels of choline. Low choline was defined as 1 SD below the mean, and high choline was defined as 1 SD above the mean. Sample sizes for each group were as follows: low choline, *n* = 9; mean choline, *n* = 41; and high choline, *n* = 10. The embedded graph shows the 95% CIs for the slope of the latency to peak amplitude at left frontal sensors. The slope outside the dotted lines is significant. CI: confidence interval; DHA: docosahexaenoic acid; SD: standard deviation.

**Figure 3:**
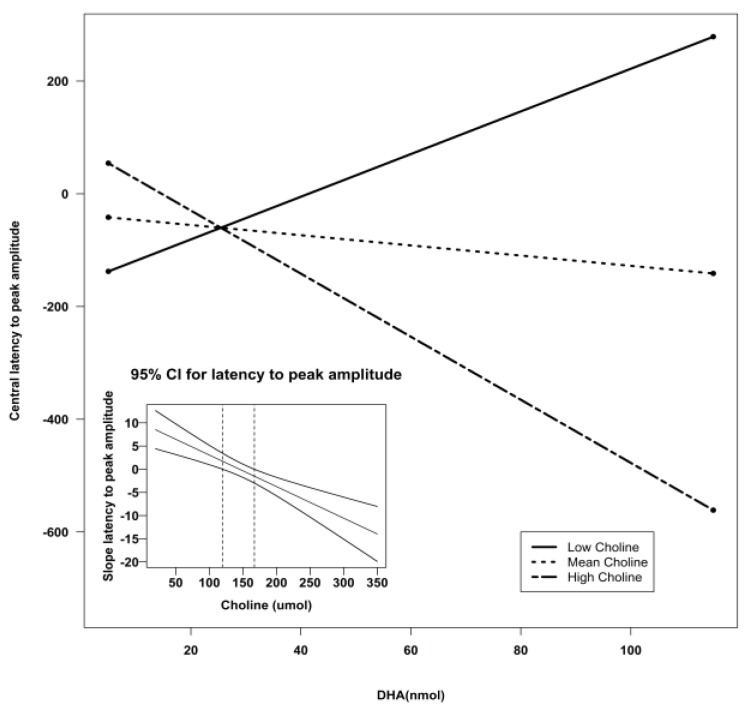
Simple slopes model of DHA by choline interaction for latency to peak amplitude at the central sensors. Negative numbers are indicative of better recognition. The figure depicts the effect of DHA at high, mean, and low levels of choline. Low choline was defined as 1 SD below the mean, and high choline was defined as 1 SD above the mean. Sample sizes for each group were as follows: low choline, *n* = 9; mean choline, *n* = 41; and high choline, *n* = 10. The embedded graph shows the 95% CIs for the slope of the latency to peak amplitude at central sensors. The slope outside the dotted lines is significant CI: confidence interval; DHA: docosahexaenoic acid; SD: standard deviation.

**Figure 4:**
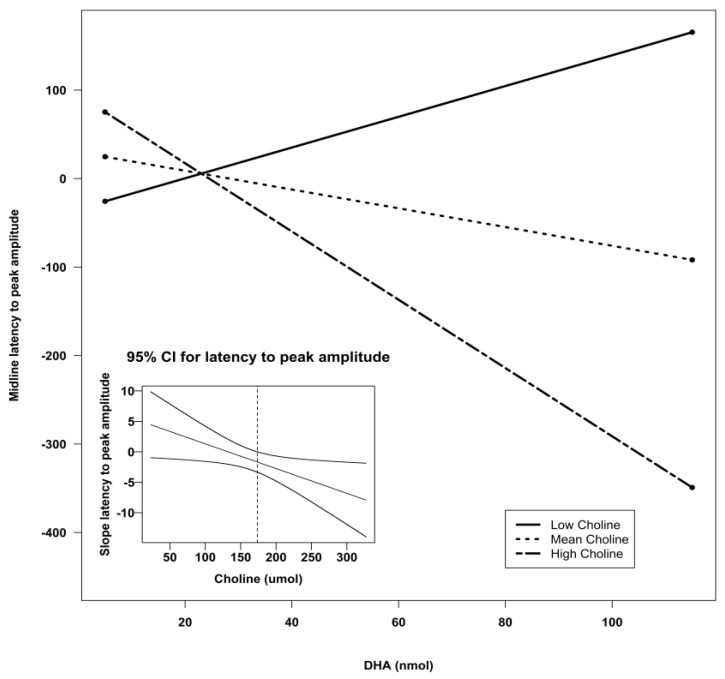
Simple slopes model of DHA by choline interaction for latency to peak amplitude at the midline sensors. Negative numbers are indicative of better recognition. The figure depicts the effect of DHA at high, mean, and low levels of choline. Low choline was defined as 1 SD below the mean, and high choline was defined as 1 SD above the mean. Sample sizes for each group were as follows: low choline, *n* = 9; mean choline, *n* = 41; and high choline, *n* = 10. The embedded graph shows the 95% CIs for the slope of the latency to peak amplitude at midline sensors. The slope to the right of the dotted lines is significant. CI: confidence interval; DHA: docosahexaenoic acid; SD: standard deviation.

Using Preacher’s interaction utility [34], significant interactions were subjected to a simple slopes analysis to investigate the nature of the associations. Figure 2, Figure 3 and Figure 4 illustrate the models of the interactions. With respect to the latency variable, difference scores that are negative are indicative of better recognition memory. Thus, the models for latency in all three clusters indicate that recognition memory would be the best when both DHA and choline are high.

**Figure 5:**
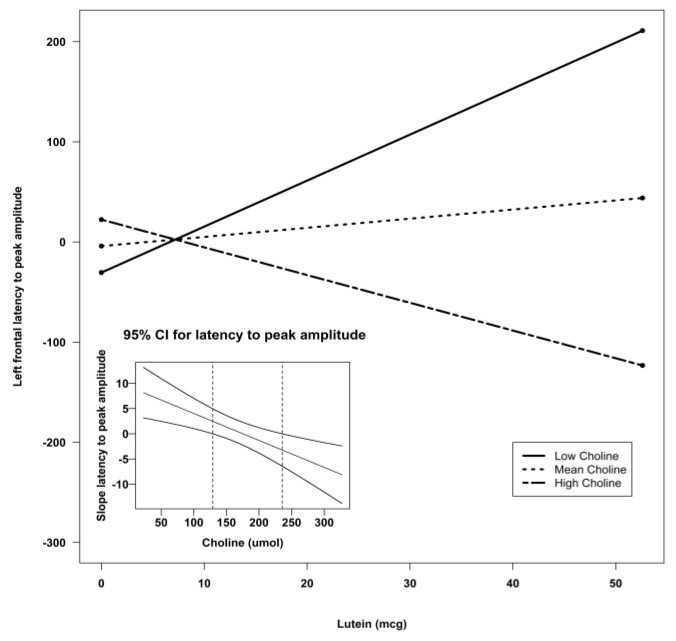
Simple slopes model of lutein by choline interaction for latency to peak amplitude at the left frontal sensors. Negative numbers are indicative of better recognition. The figure depicts the effect of lutein at high, mean, and low levels of choline. Low choline was defined as 1 SD below the mean, and high choline was defined as 1 SD above the mean. Sample sizes for each group were as follows: low choline, *n* = 9; mean choline, *n* = 41; and high choline, *n* = 10. The embedded graph shows the 95% CIs for the slope of the latency to peak amplitude at left frontal sensors. The slope outside the dotted lines is significant. CI: confidence interval; SD: standard deviation.

**Figure 6:**
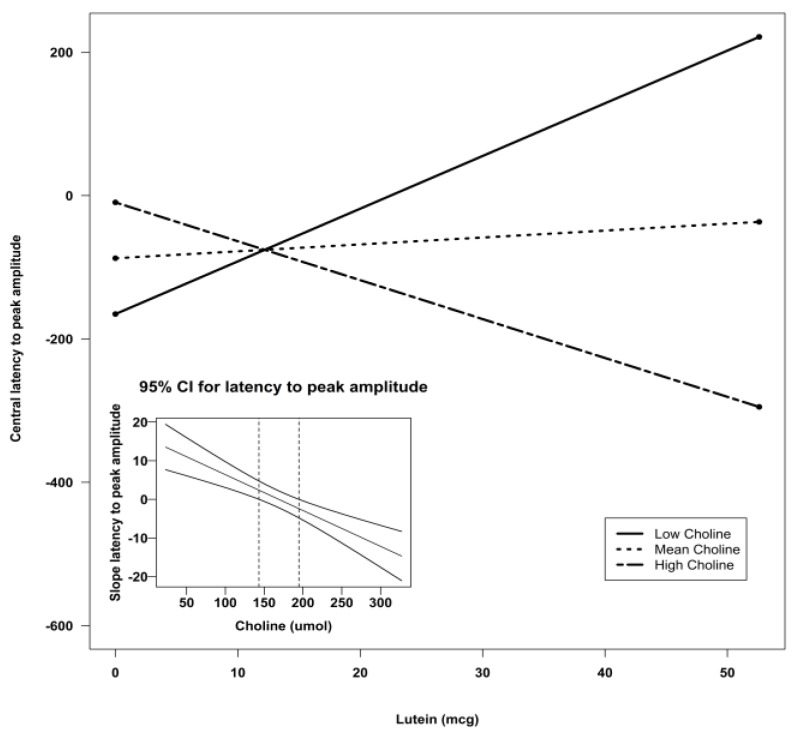
Simple slopes model of lutein by choline interaction for latency to peak amplitude at the central sensors. Negative numbers are indicative of better recognition. The figure depicts the effect of lutein at high, mean, and low levels of choline. Low choline was defined as 1 SD below the mean, and high choline was defined as 1 SD above the mean. Sample sizes for each group were as follows: low choline, *n* = 9; mean choline, *n* = 41; and high choline, *n* = 10. The embedded graph shows the 95% CIs for the slope of the latency to peak amplitude at central sensors. The slope outside the dotted lines is significant. CI: confidence interval; SD: standard deviation.

To follow up the lutein X choline interaction found in the stepwise regressions, a reduced model using choline, lutein, and their interaction to predict the outcome variables was run (Table 5). Brain activity with regard to latency was predicted at left frontal by lutein (*p* < 0.05) and the lutein X choline interaction (*p* < 0.05), which accounted for 13% of the variance in the data. At the central leads, latency was predicted by lutein (*p* = 0.001), choline (*p* < 0.05), and the lutein X choline interaction (*p* < 0.001). This model accounted for 24% Again, significant interactions were followed up using the Preacher interaction utility [34]. Figure 5 and Figure 6 illustrate the models for the lutein X choline interaction at the frontal and central leads, respectively. As negative numbers indicate better recognition memory, the model indicates that better recognition memory is seen with the combination of high choline and high lutein.

**Table 5:** Reduced model exploration of the significant interactions from the model-building analyses. Lutein, choline, and lutein X choline were used to predict the difference in latency to peak amplitude of the negative deflection seen between 200 and 900 ms when viewing novel *vs.* familiar stimuli.

Lead Grouping	Variable	β	SE	*t*-Value	*p*-Value	Model F	Model p	Model R-sq
Frontal Right	Intercept	−55.56	107.18	−0.52	0.60	1.61	0.20	0.08
	Lutein	−0.13	4.96	−0.03	0.97			
	Choline	0.72	0.59	1.23	0.22			
	Lutein X Choline	−0.02	0.02	−0.59	0.56			
Frontal Left	Intercept	−64.97	91.33	−0.71	0.48	2.60	0.06 ^	0.13
	Lutein	9.38	4.23	2.22	0.03 *			
	Choline	0.38	0.50	0.77	0.45			
	Lutein X Choline	−0.05	0.02	−2.30	0.03 *			
Central Z	Intercept	−266.55	96.55	−2.77	0.008 **	5.26	0.003 **	0.24
	Lutein	15.66	4.46	3.51	0.0009 ***			
	Choline	1.13	0.53	2.15	0.04 *			
	Lutein X Choline	−0.09	0.02	−3.78	0.0004 ***			
Midline	Intercept	−69.21	93.32	−0.74	0.46	1.71	0.18	0.09
	Lutein	7.05	4.32	1.63	0.11			
	Choline	0.42	0.51	0.82	0.42			
	Lutein X Choline	−0.05	0.02	−1.93	0.06 ^			

*** Significant at *p* < 0.001; ** Significant at *p* < 0.01; * Significant at *p* < 0.05; ^ Trending toward significance at *p* < 0.10.

## 4. Discussion

Milk nutrients (choline, lutein, and DHA) were investigated in relation to six-month-old infant recognition memory as measured by an electrophysiological assessment to test the hypothesis that these nutrients may have a synergistic effect in the brain. The results indicate that choline and DHA function together in the brain to support memory. Specifically, high choline and high DHA are associated with better recognition memory. In addition, the same association was found with choline and lutein. Importantly, these relations were evident only in the latency to peak amplitude measure and were most prominent in the central area of the scalp, but also were seen in the frontal areas and down the midline.

Latency to peak amplitude is interpreted as a measure of sustained attention [35,36,37,38,39]. Importantly, the difference between the latency to peak amplitude when viewing novel pictures and when viewing familiar pictures is assumed to be indicative of recognition memory. When infants have been exposed repeatedly to an image (familiar), they no longer have a need to process any information about the familiar image. Thus, they do not attend to it. If there is a large difference between the amplitudes when viewing novel and familiar, we assume it is because the infants *recognize* the familiar picture and do not process it, whereas they do not have a memory of the novel picture and as such, they attend to it and process it. In this study, trial unique pictures (30 unique pictures seen only once each) were used for the novel condition [30]. Traditionally, the oddball paradigm consists of one novel stimulus occurring repeatedly, but with less frequency than the familiar stimulus [40]. In previous research [30], we showed that the infants also stop processing a repeatedly presented novel picture toward the end of the session. This finding provided more support for the idea that as the image becomes familiar to the infant, processing and attention wane, and this change is evident in the latency to peak amplitude in the Nc (negative component). Thus, the difference between the latency to peak data for novel and familiar stimuli, in this paradigm, is indicative of whether or not the stimulus on the screen warranted attention and processing. From this difference, we infer recognition memory.

In this sample, recognition memory was better when DHA and choline were both high. Some researchers, but not all, who have studied the effects of DHA on cognition have observed positive results [41,42,43,44]. The effects of choline on cognition have been shown repeatedly in animal models [45]. The relation between choline and human cognition has been more elusive with one group finding effects of choline in an adult population [7], but in children, no effects of concurrent choline on cognition have been found [3] and relations between early cognition and maternal choline have been mixed [2,4,5,6]. To our knowledge, the synergistic effects of DHA and choline have not been studied previously.

It has been suggested that DHA and lutein are transported to the brain most readily by high-density lipoproteins [23], which are rich in phosphatidylcholine. Phosphatidylcholine (PC) is a phospholipid that serves as a structural component in the greater percentage of cells (50–70% of cells) as it comprises the cell membrane and influences membrane fluidity with downstream effects on signal transduction. Synthesis of PC through the hepatic *de novo* pathway by phosphatidylethanolamine N-methyltransferase (PEMT), using S-adenosylmethionine (SAM) as substrate, results in PC that is enriched in long-chain polyunsaturated fatty acids such as DHA. Importantly, PC serves an active role in mediating hepatic export of fatty acids (e.g., DHA). Erythrocyte PC-DHA is integral to our consideration of a potential mechanism behind our results as the increase of PC-DHA has been shown to be related to an increase in DHA in brain tissue in an animal model [46]. Thus choline is important in exporting hepatic DHA to extra-hepatic tissues (e.g., brain). Indeed, it has been demonstrated in healthy adult women that providing supplemental choline in the diet can increase the erythrocyte PC-DHA proportion possibly via increased hepatic PC synthesis by PEMT and its export [47]. Thus, it is reasonable to postulate that when both DHA and choline intakes are higher, more hepatic DHA is available for extra-hepatic tissues such as brain via PEMT-PC mediated export, and subsequently, DHA is likely increased in the brain relative to when the two nutrients are at lower levels. In the brain, the sequence of events when the nutrients are higher would mean that information could flow more readily across the synapses, thereby improving information processing, as seen in this study, and possibly, improving the storage of that information into memory (not assessed in this study). However, it is important to note that the interactions found to be significant in this study were with free choline not phosphatidylcholine.

Another possibility for the synergistic activity of DHA and choline could be an increase in synaptogenesis [48]. Wurtman posits that a combination of DHA, choline, and uridine is integral to synaptogenesis, and has shown in an animal model that the proteins needed for the formation of a synapse [49] are indeed increased as is dendritic complexity [50] when these compounds are supplemented. In addition, this research group has shown that the administration of DHA, choline, and uridine resulted in cognitive enhancement in the animals as evidenced by improved scores on tasks such as the Morris Water Maze [51]. Importantly, uridine is not bioavailable in foods, except human milk. Early on in human life, there is a natural proliferation of synapses [52] followed by selective retraction in a “what you don’t use, you lose” scenario. DHA, choline, and bioactive uridine (uridine monophosphate) in human milk may work to insure that proliferation.

Even though in this study maternal diet was not assessed, the nutrient content of the milk may be considered an approximation, or at a minimum, a hint at general maternal diet [53,54]. Mothers with high choline and high DHA in their milk were either eating a diet rich in these nutrients or their precursors, or they were consuming quality supplements during the breastfeeding period. If we assume that maternal diet was similar in the prenatal months, the prenatal diet of mothers with subsequent high choline and DHA milk content most likely would have resulted in an intrauterine environment that included optimal amounts of DHA and choline. Evidence in animal models [55,56,57,58] and some in studies with humans [4,44] would certainly indicate an improvement in neural structures when the fetal brain develops in a milieu that includes high levels of DHA, choline, and other nutrients such as lutein.

The choline and lutein interaction was also significant for latency to peak amplitude: when lutein and choline were high, performance was better. Lutein has been shown to be important at the eye [17,59], but has been studied mostly in elderly as it is integral to the prevention of macular degeneration. However, the level of lutein in the macula can be used as a proxy for brain tissue levels and in fact, lutein has been related to cognitive function in the elderly [19]. During development, lutein accretion in the macula may improve visual acuity in the infant. If high milk lutein results in visual acuity that develops more rapidly than in infants consuming lower lutein milk, the former may have a cognitive advantage. Seeing clearly sooner could enable infants to learn more quickly.

In addition, it is possible lutein provides as yet undiscovered support in the brain. Using metabolomics, Lieblein-Boff *et al.*, have shown that lutein not only is concentrated in the neural substrates that underlie cognition (*i.e.*, occipital, temporal, and frontal lobes), but also is correlated with fatty acids and phospholipids in the brain [60]. Carmichael and colleagues found that lutein intake is related to a lower risk of neural tube disorders [61], a risk previously thought to be attributable to low levels of folate and choline. Certainly, lutein appears in natural foods alongside choline and DHA, which also suggests co-activity. The connection between choline and lutein warrants further investigation as does the contribution of lutein itself to brain function.

Future research will also need to include investigations of maternal diet, a limitation in the work reported here. Knowing whether the diets of these mothers with high milk DHA and choline included preformed nutrients, precursor nutrients, supplements, or a combination would provide clues as to the pathways involved in the optimization of neural substrate development and/or neuronal function. Interestingly, the results of the Carmichael study [61] showed a stronger association between a lower risk of neural tube defects and higher lutein when the mothers were not taking supplements relative to when supplements were consumed. This intriguing finding implies that supplement intake may affect the bioavailability of other natural nutrients.

Just as we cannot be certain about prenatal effects, another limitation of this work is the lack of concurrent cognition and milk nutrient measures. We cannot draw any firm conclusions about how day-to-day nutrition is affecting brain activity because the nutrients were measured in one sample when the infants were three to four months old (mo) and recognition memory was measured when they were six months of age. With the exception of total choline, which reportedly is maintained at a relatively steady level in milk across the first year of life [62], nutrient levels in human milk change across the first weeks of life as the milk changes from colostrum (0–3 days) to transitional milk (5 days to 2 weeks) to mature milk (4–6 weeks to end of lactation). Indeed, DHA decreases in the first 12 weeks, but then is present at a constant level to at least six months of age [63]. Thus, the level of nutrients in milk at 3.5 mo would most likely be similar at six months if the maternal diet remained constant. Nonetheless, we have no direct evidence. Similarly, we have no evidence that the relations seen were a direct effect of improved recognition memory as opposed to a secondary effect of improved visual acuity. The analyses reported here are secondary analyses of data from a larger study. Future work will be specifically designed to clarify whether the nutrients we measured were acting as a proxy for prenatal or concurrent nutrients or if the effect we report is the result of early nutrition affecting later cognitive abilities. Future studies should also be designed to assess visual acuity.

## 5. Conclusions

The finding that choline and DHA as well as choline and lutein are working together in support of infant brain development and subsequent cognitive development is important. Since both lines of inquiry (choline and DHA in isolation) have been fraught with mixed results, a different approach is warranted. Studying the nutrients together may yield more consistent results, but also, it may provide better substantiation of the mechanism behind nutritional support of brain development. Follow-up of this cohort is underway to determine if the difference in memory abilities was merely a short-lived delay or if the effects related to differential nutrient intake was longer lasting.
